# The Erythrocyte Membrane Lipidome of Healthy Dogs: Creating a Benchmark of Fatty Acid Distribution and Interval Values

**DOI:** 10.3389/fvets.2020.00502

**Published:** 2020-08-21

**Authors:** Paraskevi Prasinou, Paolo E. Crisi, Chryssostomos Chatgilialoglu, Morena Di Tommaso, Anna Sansone, Alessandro Gramenzi, Benedetta Belà, Francesca De Santis, Andrea Boari, Carla Ferreri

**Affiliations:** ^1^Faculty of Veterinary Medicine, Veterinary Teaching Hospital, University of Teramo, Teramo, Italy; ^2^ISOF, Consiglio Nazionale delle Ricerche, Bologna, Italy

**Keywords:** membrane fatty acid, lipidomic profiles, red blood cell lipidome, healthy dog membrane profile, fatty acids cluster

## Abstract

Molecular-based approaches are rapidly developing in medicine for the evaluation of physiological and pathological conditions and discovery of new biomarkers in prevention and therapy. Fatty acid diversity and roles in health and disease in humans are topical subjects of lipidomics. In particular, membrane fatty acid-based lipidomics provides molecular data of relevance in the study of human chronic diseases, connecting metabolic, and nutritional aspects to health conditions. In veterinary medicine, membrane lipidomics, and fatty acid profiles have not been developed yet in nutritional approaches to health and in disease conditions. Using a protocol widely tested in human profiling, in the present study erythrocyte membrane lipidome was examined in 68 clinically healthy dogs, with different ages, sex, and sizes. In particular, a cluster composed of 10 fatty acids, present in membrane glycerophospholipids and representative of structural and functional properties of cell membrane, was chosen, and quantitatively analyzed. The interval values and distribution for each fatty acid of the cluster were determined, providing the first panel describing the healthy dog lipidomic membrane profile, with interesting correlation to bodyweight increases. This molecular information can be advantageously developed as benchmark in veterinary medicine for the evaluation of metabolic and nutritional status in healthy and diseased dogs.

## Introduction

**Figure 1:**
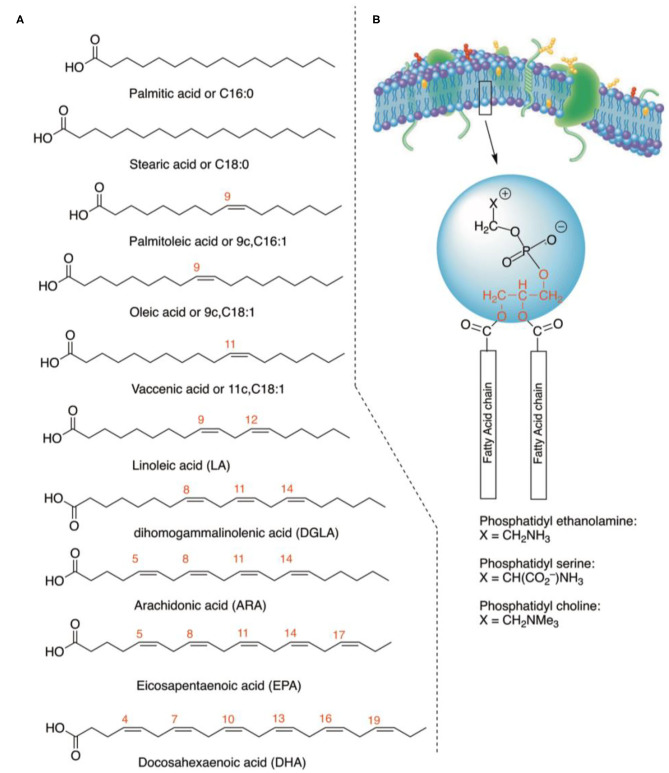
**(A)** The structure of fatty acids considered in the present work. Together with the common names, the abbreviations describe the position and geometry of the double bonds (e.g., 9c for palmitoleic acid), as well as the notation of the carbon chain length and total number of double bonds (e.g., C18:1). For PUFA in the parentheses, their acronyms are indicated. **(B)** Molecular structures of glycerophospholipids with the two fatty acid residues at the C-1 and C-2 positions of the glycerol moiety (in red), whereas in the C-3 position the polar head group is connected (examples of polar heads: ethanolamine, serine, and choline).

Lipidomics is the discipline that gathers lipids, not only considering lipid structures and their transformations, but also providing a “dynamic” interpretation of lipid diversity and functions in view of cellular, metabolic, and environmental conditions influencing living organisms (1). Investigations in lipidomics encompass a broad range of topics, going from physiological to oxidative, and pathological processes (2, 3), and large sets of data can be acquired, obtaining the map of the entire lipidome and its variations as a fingerprint of health, age, or sex status (4, 5). The development of shotgun lipidomics accelerated the process of gathering information on thousands of lipid molecules in different organisms and conditions, going from yeast, virus, and bacteria to murine models and humans (6–9). In particular, membrane lipidome has a central place in a multidisciplinary context of biophysical, biological, pharmacological, and clinical studies, which testify to its importance in fundamental processes such as cell formation, regeneration, and metabolic regulation (10–12). Focusing on membrane glycerophospholipids, the analysis of the two fatty acid chains esterified to the glycerol moiety led to the discovery of positional and geometrical isomers and of the homeostatic balance created by these hydrophobic molecules, based on the fact that the fatty acid structures influence bilayer thickness, permeability, fluidity, and membrane protein functions (10–14). In Figure 1, the main fatty acid types are shown, that can be saturated (SFA), monounsaturated (MUFA), and polyunsaturated fatty acids (PUFA). Application of fatty acid-based membrane lipidomics to human health is a hot topic of research, comparing healthy controls with disease subjects (15–18). Fatty acid analysis can be carried out starting from a non-invasive blood withdrawal, separating blood fractions that contain molecular information at different metabolic levels (18, 19). In fact, while plasma informs on the daily fatty acid dietary intakes, cell membrane fatty acids result from more complex contributions of physiological and metabolic processes together with nutritional intakes. Erythrocytes are the most numerous cell type in blood (20), and their membrane homeostasis derives from the distribution of the various fatty acid types, playing roles in several functions, such as for example oxygen distribution. Having lost DNA very early, red blood cell (RBC) fatty acids cannot come from biosynthesis but derive from exchange with lipoproteins and tissues. In this scenario, it is worth mentioning that eukaryotic cells need essential fatty acids (EFA), such as omega-3 and omega-6 PUFA, that must be taken daily from the diet in order to be processed enzymatically in the body and provide long-chain PUFAs. The PUFA pathways, together with SFA and MUFA, contribute to the formation of the cellular lipid pool and subsequently to the specific fatty acid biodistribution in RBC membranes as well as in the various organs. The RBC fatty acid composition is informative of levels reached in tissues, like muscle, and liver, and also in tissues not withdrawable during life, like retina, and brain (21–24). In human studies, we contributed to research in membrane fatty-acid-based lipidomics, making the arbitrary choice of analyzing a specific fatty acid cohort of erythrocyte membrane glycerophospholipids, made of 10 fatty acids representative of SFA, MUFA, and PUFA families. Their interval values in healthy cohorts were reported in membrane fatty acids by several studies, also by us (25–27), and in one of the most complete meta-analysis appeared in the literature (28). Taking into account that novel fatty acid pathways and transformations are continuously studied (29), it is important to make the strategic choice of a specific number of fatty acids considering their fundamental biochemical/biological roles and the consensus reached about their levels in cell membranes.

By these premises, we considered whether analogous data are reported in domestic animals, finding only a few studies on plasma or other tissues (brain, sperm) and on correlations between dietary uptake and lipidomic profiles of dogs (30–34). Limited studies presented a small number of samples (35, 36), focusing for example on PUFA (omega-6 and omega-3), and not deepening the different fatty acid families, which are instead important for molecular contribution to membrane homeostasis (37). Although fatty acids are recognized to be crucial for all animals (38), an effort to build up the methodology for fatty-acid-based membrane lipidomics in the veterinary field is missing. In the present work, we evaluated the composition of fatty acids in erythrocyte membrane phospholipids in a group of clinically healthy dogs (*n* = 68), focusing on the interval ranges of the 10 fatty acid cohort previously used for humans. We considered also correlations with dog characteristics such as age and bodyweight. The general aim of this work is to create a benchmark of fatty acid interval values in the membrane lipidome of healthy animals, useful to start a systematic approach for the examination of metabolic and nutritional status in healthy and diseased dogs.

## Materials and Methods

For the list of materials and providers see Supplementary Information.

### Inclusion Criteria and Samples Collection

Inclusion criteria for the study were: healthy dogs without any clinical or pathological evidence of disease accordingly to unremarkable history, physical examination and results of CBC, serum biochemistry, and voided urine analysis (Supplementary Table 1) within the reference range for each dog. Furthermore, recruited dogs were on commercial diet and did not receive supplements and medications in the previous 4 months (except regular preventive treatments for ecto- and endoparasites and prophylactic vaccination), as ascertained by an interview with the owners. Supplementary Table 2 presents the main dog characteristics. Blood samples (1 mL each) from healthy dogs recruited from Italian owners were collected in Ethylenediaminetetraacetic acid as tripotassium salt (EDTA) tubes from the medical staff of the Veterinary Teaching Hospital (VTH) of Teramo.

### Ethical Statement

The project has been approved by the Health Ministry and the Committee on Animal Research and Ethics of the Universities of Chieti-Pescara, Teramo and Experimental Zooprophylactic Institute of AeM (CEISA), Protocol UNICHD12 n. 1168.

### Isolation of Phospholipids From Erythrocyte Membranes and Transesterification Procedure

**Scheme 1:**
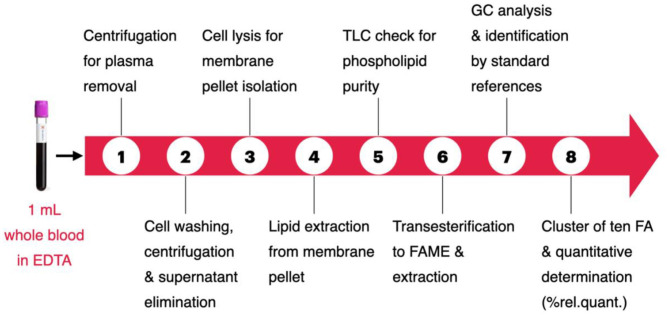
The eight steps of the fatty acid-based erythrocyte membrane lipidomic analysis. Details in the Experimental section.

The steps of the protocol procedure are depicted in Scheme 1. Step 1: separation of red blood cells from plasma, effected on 1 mL of fresh EDTA-treated whole blood sample, by two consecutive centrifugations (3,000 g × 5 min, each) followed by plasma removal. Step 2: cell washings (two times) with phosphate buffer (0.5 mL) followed by centrifugation (3,000 g × 5 min, each) and elimination of supernatant. Step 3: lysis of erythrocytes by twice mixing cells with distilled water (1 mL), followed by centrifugation (15,000 g × 15 min), in order to eliminate the aqueous layers and to obtain the erythrocyte membrane pellet. Step 4: the pellet was added by a mix of 2:1 chloroform:methanol (2 mL) and partitioned with pure water (1 mL) according to the Folch's procedure (39), for the lipid extraction. The organic layer was separated and evaporated under vacuum to dryness. Step 5: thin layer chromatography (TLC) using chloroform/methanol/water 65:25:4 to determine the purity of the phospholipid fraction (40). Step 6: the phospholipid extract was transesterified at room temperature for 10 min with 0.5 M KOH/MeOH to obtain the fatty acid methyl esters (FAMEs), derived from the fatty acid residues present in membrane glycerophospholipids. This chemical procedure avoids oxidative and degradation reactions and gives the fatty acid composition, which represents that of the membrane, as ascertained using appropriate MUFA and PUFA internal standards. FAMEs were extracted by partition between *n*-hexane (2 mL) and water (0.5 mL), followed by evaporation of the organic phase under vacuum to dryness. Steps 7 and 8: analysis using gas chromatography (GC) as described below.

### GC Analysis of FAME

First, GC analysis of the commercially available reference standard materials for the 10 fatty acids of the cluster was performed as described in Supplementary Information (Step 7 of Figure 1). Calibration curves were obtained for the quantitative analysis of each peak of the chromatogram and are shown in Supplementary Figure 1. Step 8: the FAME mixture obtained from the erythrocyte membrane pellet was dissolved in 20 μL of n-hexane and 1 μL was directly injected to the Agilent 7890B GC (Agilent, Milan) system equipped with a flame ionization detector and a (50%-cyanopropyl)-methylpolysiloxane capillary column (60 m, 0.25 mm i.d., 0.25 μm film thickness) (DB-23, Agilent; Milan). The initial oven temperature was 165°C, held for 3 min, followed by an increase of 1°C/min up to 195°C, held for 40 min, followed by a second increase of 10°C/min up to 240°C, held for 10 min. The carrier gas was hydrogen, held at a constant pressure of 16.482 psi. FAMEs were identified by comparison with the retention times of standard references (see Supplementary Figure 2 for a representative chromatogram) in agreement with previously reported procedures, satisfactorily separating all the 10 fatty acids, without superimposition of other peaks (15, 16, 26, 29). The group of 10 fatty acids corresponds to chromatographic peak areas >97%, and consisted of: two SFAs (palmitic and stearic acids); three MUFAs (palmitoleic, oleic, and cis-vaccenic acids); three PUFA omega-6 (linoleic, dihomogamma linolenic, arachidonic acids); and two PUFA omega-3 (eicosapentaenoic and docosahexaenoic acids). Using the calibration curves, the quantitative values for each peak were obtained as μg/mL, allowing to calculate also the total fatty acid contents (total SFA, total MUFA, total PUFA), ratios between the families (SFA/MUFA, omega-6/omega-3), and three indexes [unsaturation index (UI), peroxidation index (PI), PUFA balance]. Table 1 reports the interval values and the median of each value taking into account that each fatty acid is expressed as % of its quantity (% quant. rel.) respect to the sum of the 10 fatty acid quantities taken as 100%. Table 2 reports such data divided according to the sex of the dogs as median value ± standard deviation (sd).

**Table 1:** Interval values of the 10 fatty acids cohort expressed as relative percentages of the μg/mL quantities detected by gas chromatographic analyses^a^ after isolation and workup of erythrocyte membrane glycerophospholipids of healthy dogs (*n* = 68).

**Fatty acids^a^**	**Interval values^b^ (min–max)**	**Median value**
C16:0—palmitic acid	8.2–25.8	15.4
C16:1—palmitoleic acid	0.08–0.55	0.25
C18:0—stearic acid	15.6–27.3	20.2
9c,C18:1—oleic acid	6.9–14.3	9.2
11c,C18:1—vaccenic acid	1.2–2.7	1.95
LA omega-6—C18:2—linoleic acid	9.2–21	14.5
DGLA omega-6—C20:3 dihomogammalinolenic acid	0.4–2.3	1.26
ARA omega-6 C20:4—arachidonic acid	17.5–43.7	35
EPA omega-3—C20:5—eicosapentaenoic acid	0.2–1.5	0.66
DHA omega-3—C22:6—docosahexaenoic acid	0.2–2.5	0.9
Total SFA^c^	27.8–43	35.4
Total MUFA^d^	8.7–16.7	11.6
PUFA omega-3^e^	0.5–4	1.75
PUFA omega-6^f^	34.9–60.8	51
Total PUFA^g^	32.9–61.8	53
SFA/MUFA^h^	2–3.9	3
Omega-6/omega-3 ratio^i^	12.5–83.7	28.4
PUFA balance^j^	0.9–7.4	3.3
UI^k^	130–234	194.8
PI^l^	101–214	171.1

**Figure 2:**
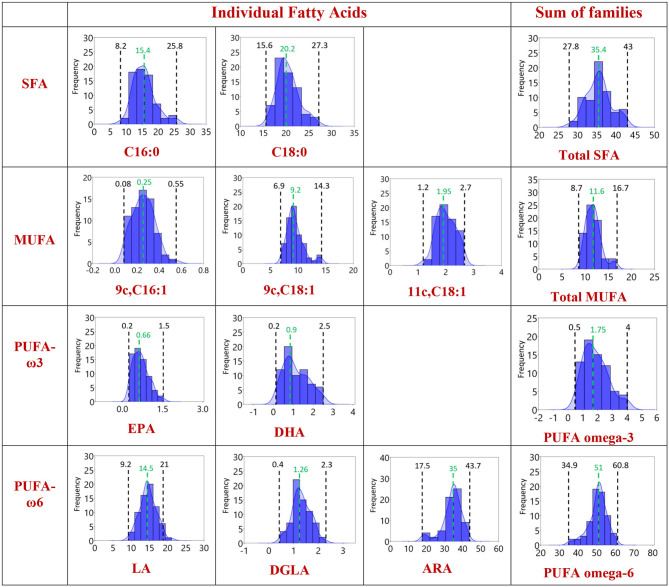
Distribution of the values in the population of Healthy Dogs (*n* = 68) for the individual fatty acids and the corresponding families using the data in Tables 1, 2 with 95% confidence interval. Each member of the fatty acid family is given in a row, the last column being the sum of the corresponding fatty acid family. Black: the minimum and the maximum value obtained. Green: the median value.

**Figure 3:**
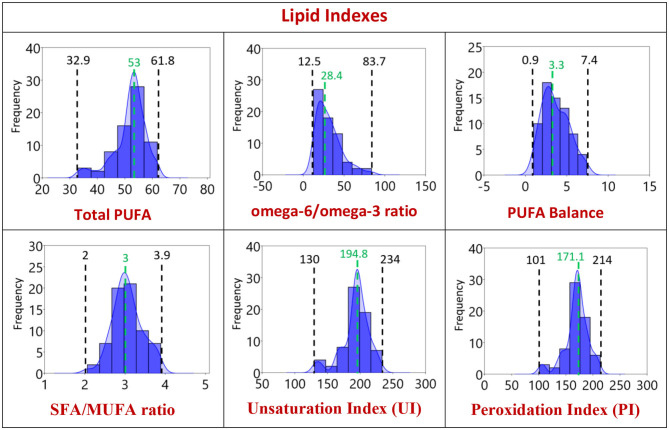
Distribution of the lipid indexes of the population of Healthy Dogs (*n* = 68) with 95% confidence interval as reported in Tables 1, 2. 1st row: total PUFA, omega-6/omega-3 and SFA/PUFA ratios; 2nd row: SFA/MUFA ratio, unsaturation and peroxidation indexes. Black: the minimum and the maximum value obtained. Green: the median value.

*The corresponding values of fatty acid families and lipid indexes are also reported*.

a *Fatty acids are evaluated as FAME (fatty acid methyl esters) after membrane isolation, lipid extraction, and derivatization as described in the Experimental section. Structures are shown in Figure 1*.

b *The values are expressed as percentage of the found quantities (calculated as μg/mL) from the gas chromatographic analysis, using calibration and quantitation protocols and standard reference compounds for each FAME, as described in Supplementary Information. The GC peak areas of the 10 fatty acids cohort corresponds to ca. 97% of the total peak areas of the chromatogram. The minimum and maximum values obtained from the population of the healthy dogs are shown for each FAME, together with the median value and the corresponding distributions as shown in Figures 2, 3*.

c *Total saturated fatty acids (SFA) = % C16:0 + % C18:0*.

d *Total monounsaturated fatty acids (MUFA) = % C16:1 + % 9c, C18:1 + % 11c, C18:1*.

e *Polyunsaturated fatty acids (PUFA) omega-3 = %EPA + %DHA*.

f *PUFA omega-6 = %LA + %DGLA + %ARA*.

g *Total PUFA = %LA + %DGLA + %ARA + %EPA + %DHA*.

h *SFA/MUFA = (% C16:0 + % C18:0)/(% C16:1 + % 9c,C18:1 + % 11c,C18:1)*.

i *Omega-6/omega-3 ratio = (%LA + %DGLA + %ARA)/ (%EPA + %DHA)*.

j *PUFA balance = [(%EPA + %DHA)/Total PUFA] × 100*.

k *UI = (%MUFA × 1) + (%LA × 2) + (%DGLA × 3) + (%ARA × 4) + (%EPA × 5) + (%DHA × 6)*.

l *PI = (%MUFA × 0.025) + (%LA × 1) + (%DGLA × 2) + (%ARA × 4) + (%EPA × 6) + (%DHA × 8)*.

**Table 2:** Cohort of 10 fatty acids expressed as percentages of the found μg/mL quantities, detected by gas chromatographic analyses of the fatty acid methyl esters (FAME)^a^ after isolation and workup of erythrocyte membrane glycerophospholipids, of male dogs (*M* = 30) and female dogs (*F* = 38).

**Fatty acids^a^**	**M (μg/mL %)^b^*n* = 30**	**F (μg/mL %)^b^*n* = 38**	**M vs. F *p*-value^c^**
C16:0—palmitic acid	15.88 ± 3.52	15.38 ± 3.53	0.5639
C16:1–palmitoleic acid	0.35 ± 0.27	0.24 ± 0.10	*0.0266*
C18:0—stearic acid	19.62 ± 2.45	21.09 ± 2.23	*0.0126*
9c,C18:1–oleic acid	10.07 ± 3.31	9.62 ± 1.85	0.4793
11c,C18:1—vaccenic acid	1.93 ± 0.35	2.06 ± 0.33	0.1209
LA omega-6—C18:2—linoleic acid	15.29 ± 2.65	14.11 ± 1.84	*0.0344*
DGLA omega-6—C20:3 dihomogammalinolenic acid	1.36 ± 0.35	1.28 ± 0.39	0.4075
ARA omega-6 C20:4—arachidonic acid	33.65 ± 7.06	34.33 ± 5.25	0.6506
EPA omega-3—C20:5—eicosapentaenoic acid	0.76 ± 0.39	0.70 ± 0.32	0.4933
DHA omega-3—C22:6—docosahexaenoic acid	1.10 ± 0.61	1.20 ± 0.67	0.5296
Total SFA^c^	35.50 ± 4.60	36.46 ± 4.12	0.3697
Total MUFA^d^	12.35 ± 3.36	11.92 ± 1.84	0.5062
PUFA omega-3^e^	1.86 ± 0.89	1.90 ± 0.83	0.8450
PUFA omega-6^f^	50.29 ± 6.80	49.72 ± 5.16	0.6944
Total PUFA^g^	52.15 ± 6.84	51.62 ± 5.30	0.7194
SFA/MUFA ratio^h^	2.98 ± 0.48	3.09 ± 0.36	0.2544
Omega-6/omega-3 ratio^i^	34.33 ± 19.32	32.78 ± 18.96	0.7423
PUFA balance^j^	3.60 ± 1.67	3.69 ± 1.55	0.8229
Unsaturation Index (UI)^k^	191.98 ± 25.47	192.00 ± 20.24	0.9977
Peroxidation Index (PI)^l^	166.26 ± 29.09	168.09 ± 22.31	0.7705

*The corresponding values of fatty acid families and lipid indexes are also reported. The significance as p-values is also indicated; italic denotes significance*.

a *FAME are expressed as percentage of the found quantities from the gas chromatographic analysis using calibration and quantitation protocols and standard reference compounds for each FAME, as described in Experimental section and Supplementary Information*.

b *The values are expressed as percentage of the found quantities (calculated as μg/mL) ± sd obtained from the gas chromatographic analyses, using calibration and quantitation protocols and standard reference compounds for each FAME, as described in Supplementary Information. The GC peak areas of the 10 fatty acids cohort corresponds to ca. 97% of the total peak areas of the chromatogram*.

c *Total SFA = % C16:0 + % C18:0*.

d *Total MUFA = % C16:1 + % 9c, C18:1 + % 11c, C18:1*.

e *PUFA omega-3 = %EPA + %DHA*.

f *PUFA omega-6 = %LA + %DGLA + %ARA*.

g *Total PUFA = %LA + %DGLA + %ARA + %EPA + %DHA*.

h *SFA/MUFA = (% C16:0 + % C18:0)/(% C16:1 + % 9c,C18:1 + % 11c,C18:1)*.

i *Omega-6/omega-3 ratio = (%LA + %DGLA + %ARA)/ (%EPA + %DHA)*.

j *PUFA balance = [(%EPA + %DHA) / Total PUFA] × 100*.

k *UI = (%MUFA × 1) + (%LA × 2) + (%DGLA × 3) + (%ARA × 4) + (%EPA × 5) + (%DHA × 6)*.

l *PI = (%MUFA × 0.025) + (%LA × 1) + (%DGLA × 2) + (%ARA × 4) + (%EPA × 6) + (%DHA × 8)*.

### Statistical Methods

Statistical analysis was performed using GraphPad Prism 6.01 software (GraphPad Software, Inc., San Diego, CA). All data were evaluated using a standard descriptive statistic and reported as mean ± sd or as median and range (minimum-maximum), based on their distribution. Normality was checked graphically or using the D'Agostino Pearson test. A comparison between male and female subgroups were done using the unpaired *t*-test or the Mann-Whitney test. According to the distribution of data, Pearson or Spearman correlations were used to evaluate the relationship between fatty acids percentages or fatty acids indexes with bodyweight and age. The threshold for the statistical significance (*p*-value) was set up at 0.05. The distribution graphs were produced using Past 3.14 software (free download; Øyvind Hammer, Natural History Museum, University of Oslo).

## Results

### The Erythrocyte Membrane Lipidome in Healthy Dogs

Blood samples were collected from 68 clinically healthy dogs, including 30 males (6 neutered) and 38 females (12 sterilized), weighting from 2.6 to 43 kg, aged from 2 to 156 months (median 41). Supplementary Table 2 shows age, sex, bodyweight, and breed of the cohort. The work up of the erythrocyte membrane glycerophospholipids to obtain the corresponding FAMEs followed the procedure described in Scheme 1, and each step is detailed in the Experimental section. The FAMEs were separated, recognized, and quantified by GC following known procedures (15, 16, 26, 29) (see Materials and Methods, and Supplementary Figure 2 for a representative GC chromatogram). The cluster of 10 fatty acids contains the following molecules (see Figure 1 for their structures): palmitic (C16:0) and stearic (C18:0) acids as SFA; palmitoleic (C16:1), oleic (9c,C18:1), and vaccenic (11c,C18:1) acids as MUFA; linoleic (LA, C18:2), dihomogammalinolenic (DGLA;C20:3), and arachidonic (AA, C20:4) acids as PUFA omega-6; eicosapentaenoic (EPA, C20:5) and docosahexaenoic (DHA, C22:6) acids as PUFA omega-3. Evaluation of the 10 peaks of the GC chromatogram was performed using calibration curves (see Supplementary Figure 2), obtaining μg/mL values and then expressing each fatty acid as relative quantitative percentage (% rel. quant.) of the total quantities of the 10 fatty acids recognized and calibrated in the GC analysis. The intervals and the median values obtained for our cohort of healthy dogs are reported in Table 1, together with lipid indexes, derived from the 10 fatty acid values, as follows: omega-6/omega-3 ratio, PUFA balance (omega-3/omega-3 + omega-6), SFA/MUFA ratio, UI (UI = MUFA tot × 1 + C18:2 × 2 + C20:3 × 3 + C20:4 × 4 + C20:5 × 5 + C22:6 × 6), and PI (PI = MUFA tot × 0.025 + C18:2 × 1 + C20:3 × 2 + C20:4 × 4 + C20:5 × 6 + C22:6 × 8) (41). A few fatty acids of dog erythrocyte membranes were previously reported (35–37), and their values are listed in Supplementary Table 3.

In Figure 2, the distribution graphics for each fatty acid (% rel. quant.) are shown together with their families (SFA, MUFA, and PUFA omega-6 and omega-3), whereas Figure 3 shows the distribution graphics of the total PUFA and lipid indexes obtained from the 10 fatty acid values. Figures 2, 3 also show minimum and maximum values, as well as the median value of each measured parameter, as listed in Table 1.

### The Correlations of Fatty Acids With Dog Characteristics

We used the above described interval values to evaluate correlations between FAME types, families, and lipid indexes with the dog characteristics. In Supplementary Information, the correlation graphs are shown (Supplementary Figures 3–6). For the age the only correlation was found with eicosapentaenoic acid (EPA, omega-3; *p* < 0.001, *r* = 0.396). With bodyweight, several fatty acid parameters correlated significantly, as follows: positive correlation with palmitic acid (C16:0; *p* = 0.001, *r* = 0.385) and the total amount of SFA (*p* = 0.001, *r* = 0.402). The MUFA palmitoleic acid (C16:1) significantly correlated in a positive manner to bodyweight (*p* = 0.007; *r* = 0.326). The bodyweight negatively correlated with arachidonic acid (*p* = 0.036, *r* = 0.257), the total content of the omega-6 PUFA (*p* = 0.003, *r* = 0.361), and the total amount of PUFA (*p* = 0.004, *r* = 0.347). Moreover, both UI and PI showed negative correlation with bodyweight (*p* =0.031, *r* = 0.301 and *p* = 0.037, *r* = 0.256, respectively). A statistical analysis based on the different breeds of the healthy dogs was not possible because of the limited number of samples for each breed and in addition 24 out of the total 68 (35%) of the healthy dogs recruited for this study were mixed-breed.

Subsequently, two groups of male and female healthy dogs were examined to envisage differences of the membrane RBC fatty acid composition between the two genders. Because of the small number of sterilized females (*n* = 12) and neutered males (*n* = 6), we did not consider this additional characteristic to further discriminate groups. The values for the individual FAME and the lipid indexes for male and female healthy dogs are reported as mean ± sd values in Table 2, expressed as quantitative relative percentages (% rel. quant.) of each value over the total 10 fatty acids values, as explained above. Significant differences were found for the lower levels of stearic and linoleic acids (*p* = 0.0126 and *p* = 0.0266, respectively) and higher levels of palmitoleic acid (*p* = 0.0344) in female than in male dogs. The corresponding graphical representations are shown Supplementary Figures 7, 8.

## Discussion

For the first time, the erythrocyte membrane lipidome characterization of the fatty acid content of glycerophospholipids is provided in a cohort of clinically healthy dogs (*n* = 68). The rationale for the choice of 10 fatty acids takes into account the most representative components in terms of structural and functional roles played by such hydrophobic moieties in membrane phospholipids, as well as the nutritionally important PUFAs (see Figure 1). We arbitrarily chose this cohort for the application to veterinary analyses, taking into consideration our and others' work done in humans with physiological and pathological conditions (15–18, 25–28). It is worth underlining that studies reported in literature on fatty acids of erythrocyte membranes in both humans and animals do not report the same cohort of fatty acids. For humans, the meta-analysis reported by Hodson et al. (28) highlighted 13 SFA, MUFA, and PUFA moieties in erythrocyte membrane phospholipids obtained from nine studies of 321 men and 70 women. In another study on infants, only the total SFA and MUFA values are given without specifying each fatty acid type, whereas for the omega-6 pathway together with the C18 and C20 PUFA long-chain fatty acids such as 22:4 and 22:5 are evaluated, which are not commonly reported in other studies (21). With the emerging field of lipidomics, including our own research, new fatty acid structures are evidenced, such as for example the presence of the n-10 MUFA family, with 6*cis*-C16:1 (sapienic acid) as positional isomer of palmitoleic acid, and the corresponding geometrical trans isomer (6*trans*-C16:1). These new markers indicate the metabolic partition of delta-6 desaturase enzymes between palmitic and linoleic acids and the free radical-based transformations (11), respectively, but it is not possible to define yet their participation and effects for the membrane composition. In model animals as well as in humans, increased or decreased fatty acid levels were found in several diseases such as for example, for kidney (42) or liver diseases (43) being such alterations correlated with other biochemical markers. Newly identified fatty acid markers need further research to establish their normal and altered levels. Considering such complexity, it was evaluated of strategic importance to concentrate on a specific number of fatty acids, that is, the 10 fatty acid cohort, and on a specific cell compartment, that is, the erythrocyte membrane, in order to get a simplified, yet representative and significative, approach for the examination of known metabolic and nutritional influences on the biophysically regulated homeostatic control of membrane formation and remodeling. The cohort includes fatty acids that cannot be missed in biological membranes, that are characteristic for each tissue type (8, 14, 24, 44) and their interval values (minimum and maximum values) in the specific membrane type, such as in erythrocytes, derive from population studies, as previously mentioned.

It is worth mentioning that, when fatty acid analysis is performed, it is very important to ascertain that the subjects did not follow any supplementation during the last 4 months. We asked this information to the owners during the recruitment, and no specific supplementation or regular consumption of specifically enriched food emerged. We remark that in canine diets, it is becoming more and more popular the use of omega-3 supplements or commercial “omega-3 enriched” diets, that lead to the uptake of this EFA family. It is known that such supplementations can change the results of lipid analyses (45).

The analytical methodology is also important to ensure the efficient separation of the 10 fatty acid cohort especially of unsaturated fatty acids with the same fatty acid chain length but different geometry and position of the double bonds. In this respect, the library of geometrical and positional isomers in our hands plays an important role to check peak superimposition (11, 15, 16, 29). The overall fatty acid distribution reported in Table 1 and Figures 2, 3 highlights that in the dog erythrocyte membrane lipidome the omega-6 PUFA content is prevalent followed by SFA and MUFA. It is worth underlining that omega-3 fatty acids are present in minimal concentrations, marking the difference with human lipidome (11, 18, 24, 30–32). Moreover, in healthy dogs the SFAs, stearic and palmitic acids, and the omega-6 PUFAs, arachidonic, and linoleic acids, sum up to nearly the 94% of the total membrane RBC fatty acids, as already observed in other mammalian species (24). In the distribution graphs of Figure 2, large interval ranges for the palmitic acid percentage (8.2–25.8) and arachidonic acid (17.5–43.7) can be appreciated. It is also interesting to note that in 80% of the dogs' narrower ranges are described (palmitic acid: 8.5–20%, arachidonic acid: 30–40%). On the other hand, the distribution of linoleic acid values follows a gaussian-like behavior, with a probability distribution symmetric about the mean, showing that data near the mean are more frequent in occurrence than data far from the mean. The different distributions of SFA, C18, and C20 PUFA omega-6 indicate that it is necessary to gather large sets of these data for mathematical treatment to model their roles in the membrane compositions (46). It is also worth pointing out again that the omega-6 are EFA, not prepared directly by biosynthesis, and also dogs need the intake of linoleic acid as precursor of other biosynthetically prepared omega-6 fatty acids. The importance of ascertaining the omega-6 dietary supply to animals represents an historical highlight in veterinary medicine, having been noted in canine dermatological problems by Burr and Burr (47). Therefore, it is of extreme importance to follow up the intakes of linoleic acid and its metabolic transformations to DGLA and arachidonic acid, together with their incorporation at the level of cell membranes, since they regulate fundamental structural and functional properties for health in the whole. This information can be integrated in a panel with other biochemical, biomolecular, clinical parameters for a thorough evaluation of health in dogs.

The strength of our cohort of fatty acids is to provide a valuable set of metabolic information to examine: (1) data on the PUFA pathways that start from precursors that must be necessarily taken from the diet thus reporting on the omega-6 and omega-3 fatty acid balance, that in dogs such balance is shifted toward the omega-6 family; and (2) data on the SFA-MUFA pathway that starts from the *de-novo* biosynthesis of the first fatty acid in the body, that is, the SFA palmitic acid, thus including the effects of insulin response and liver conditions (1, 24). From these crucial data sets, the influence of enzymatic activities, hormones, and nutrients can be studied, and it is possible to use the specific SFA-MUFA-PUFA cohort to examine metabolic profiles, decide nutritional-based strategies, and follow up on the changes during physiological and pathological conditions, including oxidative stress (1–3, 9, 11). The limitations are due to the small size of our data, compared with the large data set provided by other powerful lipidomic techniques, although the balance cost-effectiveness are important parameters to evaluate in wide applications to population. Another limitation of our study is in the non-homogeneous distribution of male and female dogs and in the diversity of breeds, but the prosecution of data collection will certainly compensate such weaknesses.

The omega-6 EFA linoleic acid and its transformation to DGLA and ARA, which is predominant in dogs, affect not only from the point of view of the membrane structural properties but also affects the membrane response to stimuli. In fact, upon different kinds of stimuli, phospholipase A_2_ is activated and liberates the fatty acid moieties from the cell membrane phospholipids (48); therefore, the membrane fatty acid composition can be considered a precious information of the pro- and anti- inflammatory predisposition of the organism (49, 50). Indeed, liberation of SFA, MUFA, and PUFA fatty acids generates potent mediators, such as prostaglandins, leukotrienes, endocannabinoids, and many others that influence dog metabolism in health and diseases (51–53). There is a growing attention to the lipidomic analysis in animal studies, as reported recently for variations of phospholipids of whole blood and plasma in canine inflammatory bowel disease (53). In this context we believe that the present work on erythrocyte membrane fatty acid cohort in healthy animals could represent the benchmark for assessment of healthy dog molecular profile.

A focus of our approach in dogs was to observe the omega-3 value and its distribution in healthy dogs, since omega-3 are EFAs, and EPA and DHA levels derive from the dietary intake of the precursor (alpha-linolenic acid). It is well-known that this pathway in animals is less prevalent than omega-6 (52). Our data confirmed low levels and a narrow range (0.5–4) (Table 1) together with a significant age-related increase of EPA (Supplementary Figure 3). It is worth noting that in humans EPA and DHA levels are strongly influenced by dietary intakes, but some studies have shown that age remains a significant determinant of EPA and DHA status regardless of dietary intake (54). Omega-3 are known to have specific anti-inflammatory activities and, being the omega-6/omega-3 ratio naturally high in dogs (12-7-83.7), it is evident that their increase in the cellular environment, influencing this ratio, can exert significant metabolic effects. In this context, the establishment of omega-3 levels under various dog health conditions can be interesting to precisely individuate levels of omega-3 to introduce in the regular diets of animals.

Considering the correlations found in our work, it is worth underlining that *de novo* lipogenesis with formation of palmitic acid and palmitoleic acid are known biomarkers of bodyweight increase in animals and humans (15, 55), and their increase over the normal levels are indicators of metabolic derangements in humans at older ages (56). We found positive correlation between bodyweight and the values of palmitoleic, stearic acids, and total SFA of our healthy dog cohort. At the same time, the negative correlation between bodyweight with all unsaturated indicators (arachidonic acid, PUFA omega-6, UI, PI) indicates a shift of membrane components from unsaturated to saturated moieties, that will be worth of further deepening, especially in the dog obesity conditions. An increase of SFA and MUFA components has been correlated with suppression of inflammatory response in animal models (55).

Correlations of the fatty acid data with different breeds need more data, whereas interesting observations could be done related to dog sex. The lower levels of stearic acid and the higher levels of palmitoleic acid detected in male dogs indicate that SFA to MUFA pathway functions more than in females. Palmitoleic acid is a lipokine that travels to muscles and liver, where it improves cell sensitivity to insulin, blocks fat accumulation in the liver (57), and seems to be involved in regulating muscle mass (57, 58). Although preliminarily, this different distribution of FA indirectly indicates differences in metabolism of dogs according to the sex, to be deepened also considering larger numbers of sterilized females and neutered males.

## Conclusion

In this paper, we provided the first panel of erythrocyte membrane fatty acids in healthy dogs choosing a cohort representative for the main structural and functional roles of these hydrophobic molecules in the cell membrane compartment. The panel of 10 fatty acids represents a set of information, chosen for their biochemical, nutritional, and metabolic significance, and their reference ranges for healthy dogs can represent a benchmark to evaluate pathological conditions. It is worth to remark that the effects of fatty acids in cellular metabolism departing from the membrane release and remodeling processes are more and more studied also for immune functions (59); therefore, the knowledge of the membrane asset can be a useful indication for the examination of animal response at molecular level. Indeed, the protocol herein described, including calibration and quantitation procedures, can be applied to large animal populations, with molecular information that are ready to be combined with other medical data sets for mining techniques, which are nowadays under study for the exploitation of disease risks and prevention plans (60).

Clinically speaking, since imbalances in lipid metabolism contributes to diverse animal phenotypes and disease states, ranging from inflammation and cancer to metabolic diseases, the study of membrane lipids in normal and pathological states can successfully contribute to monitor health conditions as well as the result of treatments. Membrane lipidomic research in veterinary will provide, as done for humans, deep insights in mechanisms of several diseases, helping to identify molecular targets, patient selection for better treatment response, prediction and monitoring of treatment efficacy, and response to therapeutic measures. In the light of this, membrane lipidomics may become part of the arsenal adopted for clinical diagnostics in veterinary medicine, useful for the evaluation of a disease onset and its progression, representing a potential avenue to individualized treatment and monitoring in different pathological states.

## Data Availability Statement

All datasets generated for this study are included in the article/Supplementary Material.

## Ethics Statement

This animal study was reviewed and approved by Health Ministry and the Committee on Animal Research and Ethics of the Universities of Chieti-Pescara, Teramo and Experimental Zooprophylactic Institute of AeM (CEISA), Protocol UNICHD12 n. 1168. Written informed consent was obtained from the owners for the participation of their animals in this study.

## Author Contributions

CF, CC, and AB: conceptualization. CF, CC, AB, and PC: methodology and supervision. PC, AG, MD, BB, and FD: recruitment and clinical data. AS and PP: lipidomic analyses. CF, PC, AB, CC, and AG: data curation. CF: writing—original draft preparation. CF, CC, AB, PC, AG, and PP: writing—review and editing. CF, AB, and AG: funding acquisition. All authors contributed to the article and approved the submitted and revised versions.

## Conflict of Interest

The authors declare that the research was conducted in the absence of any commercial or financial relationships that could be construed as a potential conflict of interest.
