# Supplementary material for: The Erythrocyte Membrane Lipidome of Healthy Dogs: Creating a Benchmark of Fatty Acid Distribution and Interval Values
Source: Front Vet Sci. 2020 Aug 21;7:502. doi: 10.3389/fvets.2020.00502 (PMC7472600; doi:10.3389/fvets.2020.00502)
Supplement: Supplementary file 2 [file Data_Sheet_2.pdf]

Supplementary Material

The erythrocyte membrane lipidome of healthy dogs: creating a benchmark of fatty acid distribution and interval values

Paraskevi Prasinou[1], Paolo E. Crisi[1], Chryssostomos Chatgilialoglu[2], Morena Di Tommaso[1], Anna Sansone[2], Alessandro Gramenzi[1], Benedetta Belà[1], Francesca De Santis[1], Andrea Boari[*,1], Carla Ferreri[*,2]

[1]Veterinary Teaching Hospital, Faculty of Veterinary Medicine, University of Teramo, Teramo, Italy

[2]ISOF, Consiglio Nazionale delle Ricerche, Bologna, Italy

***Co-Corresponding Authors:**
Dr. Carla Ferreri, carla.ferreri@isof.cnr.it; Prof. Andrea Boari, aboari@unite.it

Supplementary Table 1. Complete blood count, serum chemistry and urine parameters evaluated to determine the healthy status of enrolled dogs.

**Variable (Unit of measure)**	**Manufacturers**
Red Blood Cells (cells/mm³)	ADVIA 2120; Siemens Healthcare Diagnostics
Hematocrit (%)	
Hemoglobin (g/dL)	
Mean Cell Volume (fL)	
Mean Platelet Volume (fL)	
Mean Corpuscular Hemoglobin Concentration (g/dL)	
Mean Corpuscular Hemoglobin (pgr)	
Red Blood Cell Distribution Width (%)	
White Blood Cells (cells/mm³)	
Neutrophils (cells/mm³)	
Lymphocytes (cells/mm³)	
Monocytes (cells/mm³)	
Eosinophils (cells/mm³)	
Platelets (cells/mm³)	
Basophils (cells/mm³)	
Microscopic evaluation blood smear	
Creatinine (mg/dl)	OLYMPUS AU 400;

Urea (mg/dl)	Olympus-Beckman Coulter
Total calcium (mg/dl)	
Ionized calcium (mmol/l)	
Phosphate (mg/dl)	
Sodium (mEq/l)	
Chloride (mEq/l)	
Potassium (mEq/l)	
Magnesium (mg/dl)	
Total proteins (g/dl)	
Albumin (g/dl)	
Albumin-to-globulin ratio	
Glucose (mg/dl)	
Fructosamine (mg/dl)	
Aspartate transaminase (U/l)	
Alanine transaminase (U/l)	
Gamma(γ)-glutamyl transferase (U/l)	
Alkaline phosphatase (U/l)	
Total bilirubin (mg/dl)	
Total cholesterol (mg/dl)	
Triglycerides (mg/dl)	

Lipase (U/l)	
Amylase (U/l)	
c-reactive protein (mg/dl)	
Iron (µg/dL)	
Total Iron Binding Capacity (µg/dL)	
Latent Iron Binding Capacity (µg/dL)	
Iron Saturation (%)	
Urinary Specific Gravity	Refractometer; American Optical
Urine dipstick examination	Combur10Test; Roche Diagnostic
Microscopic evaluation urine sediment	

Supplementary Table 2. Breed, sex, age and bodyweight of the recruited healthy dogs (n=68). M: male; F: female; Mn: neutered male; Fs: spayed female.

n	Breed	Sex	Age (months)	Bodyweight (k)
**1**	Boxer	M	36	30
**2**	Boxer	F	34	24,5
**3**	Mixed-breed	Fs	48	8
**4**	Boxer	F	42	22
**5**	Mixed-breed	F	63	8
**6**	Dobermann	F	40	35

n	Breed	Sex	Age (months)	Bodyweight (k)
**7**	Mixed-breed	F	75	8
**8**	Romagna Water Dog	M	38	14
**9**	Italian Mastiff	M	2	8
**10**	Romagna Water Dog	F	52	13
**11**	Mixed-breed	F	12	22
**12**	Mixed-breed	F	12	21,8
**13**	Alaskan Malamute	M	29	25
**14**	Boxer	M	36	35
**15**	Mixed-breed	Fs	96	29,5
**16**	Mixed-breed	M	98	17,5
**17**	Mixed-breed	M	6	17,5
**18**	Mixed-breed	Fs	20	24,6
**19**	Mixed-breed	Mn	26	8
**20**	Golden Retriever	M	112	25,8
**21**	Italian Mastiff	M	5	29
**22**	Mixed-breed	Mn	90	14
**23**	Mixed-breed	M	36	30,1
**24**	Mixed-breed	Mn	12	6,7
**25**	Mixed-breed	Fs	60	20
**26**	Mixed-breed	F	48	16

n	Breed	Sex	Age (months)	Bodyweight (k)
**27**	Mixed-breed	Mn	36	22
**28**	Mixed-breed	M	36	24
**29**	Border Collie	M	65	30
**30**	Mixed-breed	Fs	48	28
**31**	Breton	Mn	48	17
**32**	Rottweiler	F	9	35
**33**	Miniature poodle	F	48	2,6
**34**	Maremma Scenthound	F	24	12
**35**	Golden Retriever	Fs	156	29
**36**	Maremma Scenthound	Fs	31	16
**37**	Border Collie	F	9	10
**38**	Cocker Spaniel	F	80	15
**39**	Labrador	Mn	91	30
**40**	Pinscher	M	83	5
**41**	Mixed-breed	M	36	4
**42**	Mixed-breed	M	110	15,85
**43**	Border Collie	F	18	22
**44**	Mixed-breed	Fs	118	18
**45**	Siberian Husky	F	102	24

n	Breed	Sex	Age (months)	Bodyweight (k)
**46**	Siberian Husky	Fs	138	26
**47**	Labrador	Fs	30	28
**48**	Mixed-breed	M	117	7
**49**	Labrador	M	6	32
**50**	Labrador	M	80	43
**51**	Labrador	M	52	35
**52**	Labrador	F	122	40
**53**	Labrador	F	24	32
**54**	Labrador	F	85	35
**55**	Labrador	F	24	39
**56**	Labrador	F	24	30
**57**	Labrador	F	35	35
**58**	Labrador	F	50	35
**59**	Labrador	M	22	43
**60**	Miniature poodle	Fs	29	6
**61**	Jack Russell Terrier	M	51	7
**62**	Cocker Spaniel	M	47	14
**63**	Rottweiler	F	5	20
**64**	Mixed-breed	F	21	22,3
**65**	Miniature poodle	M	95	5

n	Breed	Sex	Age (months)	Bodyweight (k)
**66**	Siberian Husky	Fs	51	22,5
**67**	Jack Russell Terrier	M	69	9,7
**68**	Mixed-breed	F	25	29,5

Materials

The materials with the corresponding suppliers are indicated here below:

Materials	Company
n-Hexane 95%	TITOLCHIMICA, Pontecchio Polesine (Ro) Italy
Methyl alcohol HPLC	TITOLCHIMICA Pontecchio Polesine (Ro) Italy
Chloroform extra pure 99.5%	TITOLCHIMICA Pontecchio Polesine (Ro) Italy
PBS pH 7,4 RS	Carlo Erba, Milan (Italy)
Polar Lipid Mixture (quantitative)	MATREYA LLC State College, PA, USA
non-Polar Lipid Mixture B (quantitative)	MATREYA LLC State College, PA,USA
Phosphatidylserine	MATREYA LLC State College, PA, USA
L-α-Phosphatidylcholine	Merck, Darmstadt, Germany
ALUGRAM Xtra sheets 200x200mm	Carlo Erba, Milan Italy
Potassium hydroxide, pellets RPE - For analysis	Carlo Erba, Milan Italy
Sodium sulfate anhydrous RS - For anhydrification	Carlo Erba, Milan Italy
C16:0 – palmitic acid methyl ester	Merck, Darmstadt Germany
C16:1 – palmitoleic acid methyl ester	Merck, Darmstadt Germany
C18:0 – stearic acid methyl ester	Supelco, Bellefonte, PA, USA
9c, C18:1 – oleic acid methyl ester	Merck, Darmstadt, Germany
11c, C18:1 – vaccenic acid methyl ester	Supelco, Bellefonte, PA ,USA
LA omega-6 – C18:2 – linoleic acid methyl ester	Merck, Darmstadt Germany
DGLA omega-6 – C20:3 dihomogammalinolenic acid methyl ester	Merck, Darmstadt Germany

ARA omega-6 C20:4 – arachidonic acid methyl ester	Merck, Darmstadt Germany
EPA omega-3 – C20:5 – eicosapentaenoic acid methyl ester	Supelco, Bellefonte, PA, USA
DHA omega-3 – C22:6 – docosahexaenoic acid methyl ester	Merck, Darmstadt Germany
Supelco 27 component FAME mix	Supelco, Bellefonte, PA, USA

Materials were used as received.

GC analysis of FAME – Calibration procedure

For this study we chose to study a cluster of 10 fatty acids, which also corresponds to chromatographic peak areas >97%. This cluster consists of: 2 saturated fatty acids (SFA: palmitic and stearic acids); 3 monounsaturated fatty acids (MUFA, palmitoleic, oleic and cis-vaccenic acids); 3 polyunsaturated fatty acids omega-6 (PUFA, linoleic, dihomo-gamma linolenic, arachidonic acids); 2 polyunsaturated fatty acids omega-3 (PUFA, eicosapentaenoic and docosahexaenoic acids) as shown in Table 1 of the main text.

The quantitation of the fatty acids was carried out by calibration procedures, for which the following protocol has been followed:

initially a *n*-hexane (HPLC grade, Titolchimica) 5mM solution of stearic acid methyl ester (2 mg in 1340 μL) was prepared and 1μl was directly injected to the Agilent 7890B GC system equipped with a flame ionization detector and a DB-23 (50%-Cyanopropyl)-methylpolysiloxane capillary column (60 m, 0.25 mm i.d., 0.25 μm film thickness). The following oven conditions were established to be kept for all the analyses: the initial temperature was 165 °C, held for 3 min, followed by an increase of 1 °C/min up to 195 °C, held for 40 min, followed by a second increase of 10 °C/min up to 240 °C, held for 10 min. The carrier gas was hydrogen, held at a constant pressure of 16.482 psi. The injections were repeated in triplicates.

The second round of injections for calibration was then performed with 0.5 mM solution of the same fatty acid methyl ester (taking 100μL of the initial solution and diluting with 900μL of *n*-hexane), injecting 1 μL as previously described for triplicates.

The same protocol was carried out using dilutions of 0.05mM, 0.005mM and 0.0005mM of stearic acid methyl ester.

In all the injections a calibration curve was created using the software of the GC equipment (Agilent 7890B GC system, Agilent, Milan).
Using the concentration of 0.0005mM for methyl stearate, the corresponding peak area was detectable but not quantifiable, indicating this concentration as the limit of detection (LOD) of the specific GC system (<0.5nM).

The above protocol has been followed for all the fatty acids of the cohort.

Reference FAMEs				
**SFA**	**C16:0**	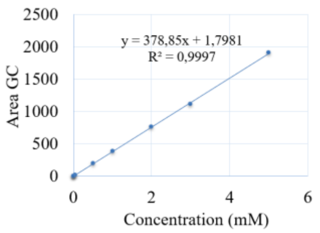	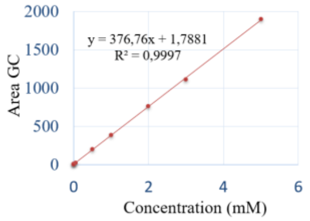	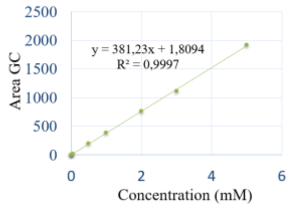
	**C18:0**	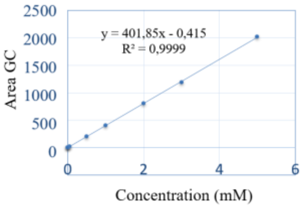	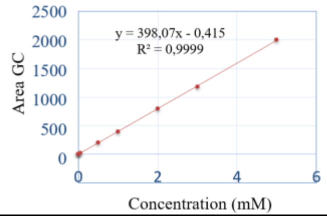	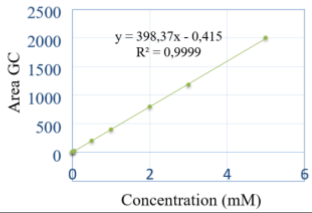
**MUFA**	**9c,C16:1**	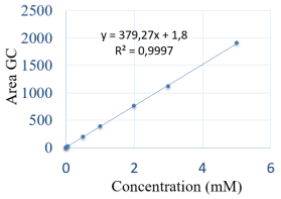	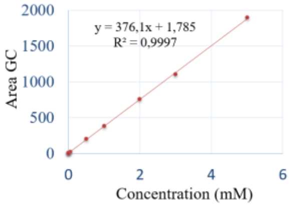	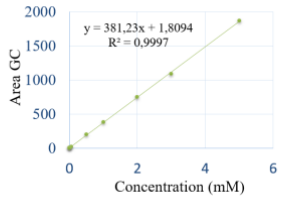
	**9c,C18:1**	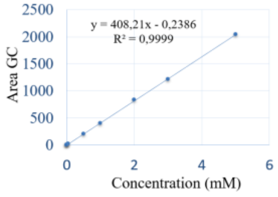	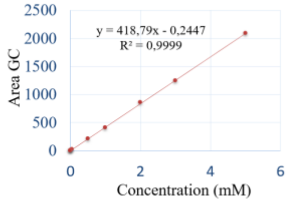	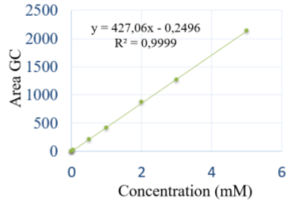
	**11c,C18:1**	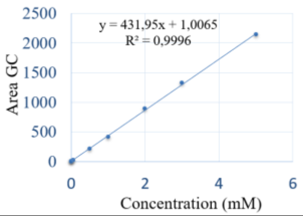	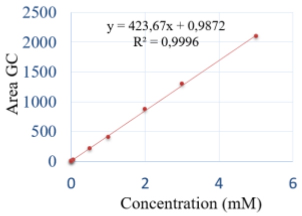	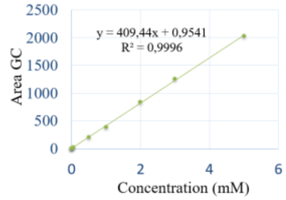
**PUFA omega-3**	**EPA**	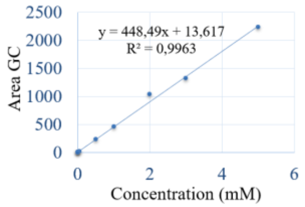	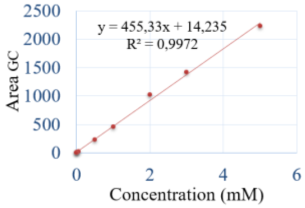	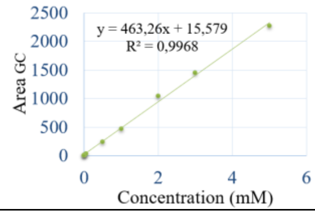

<table>
<tr><td></td><td>DHA</td><td>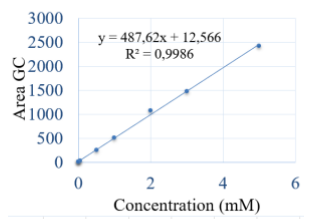</td><td>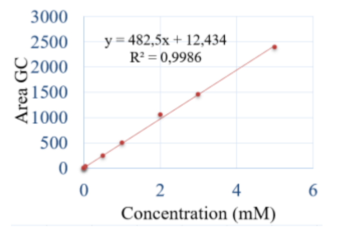</td><td>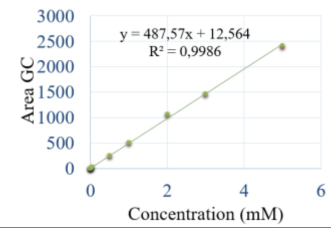</td></tr>
<tr><td rowspan="3">PUFA omega -6</td><td>LA</td><td>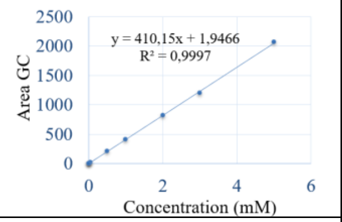</td><td>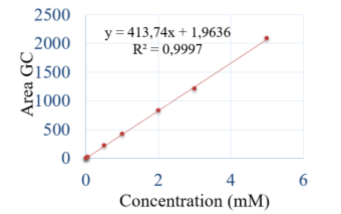</td><td>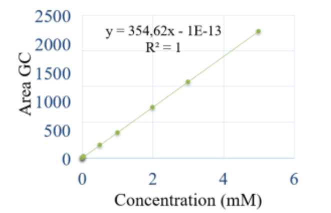</td></tr>
<tr><td>DGLA</td><td>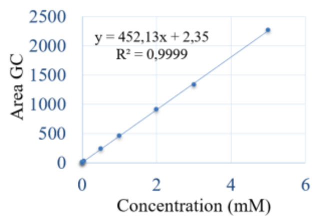</td><td>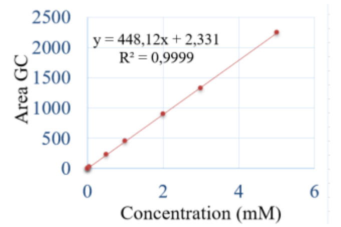</td><td>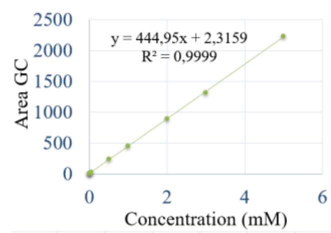</td></tr>
<tr><td>ARA</td><td>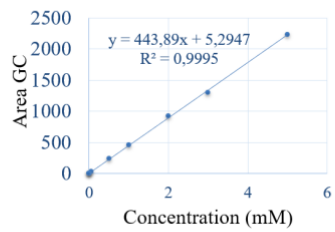</td><td>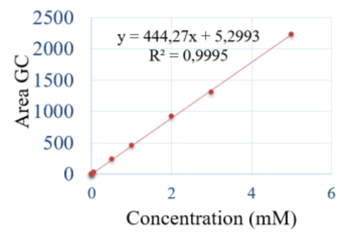</td><td>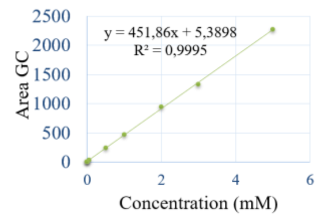</td></tr>
<tr><td colspan="5">Reference FAMEs</td></tr>
<tr><td rowspan="2">SFA</td><td>C16:0</td><td>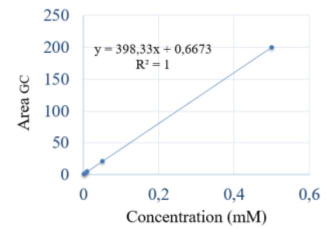</td><td>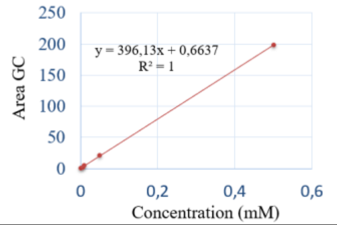</td><td>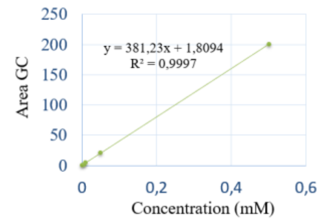</td></tr>
<tr><td>C18:0</td><td>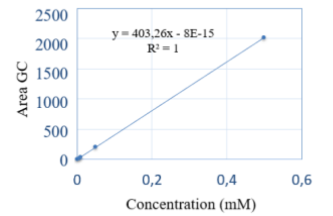</td><td>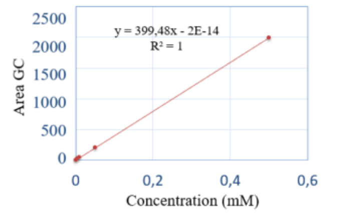</td><td>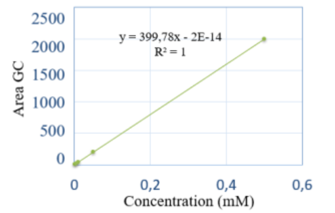</td></tr>
<tr><td>MUFA</td><td>9c,C16:1</td><td>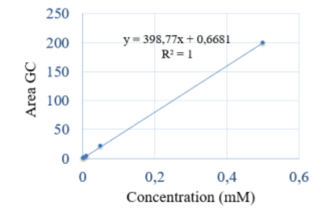</td><td>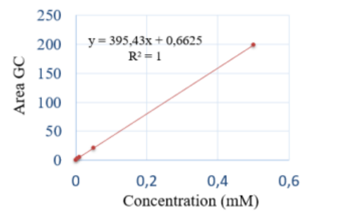</td><td>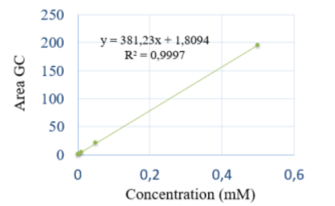</td></tr>
</table>

	**9c,C18:1**	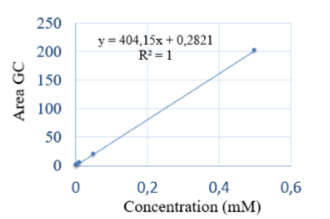	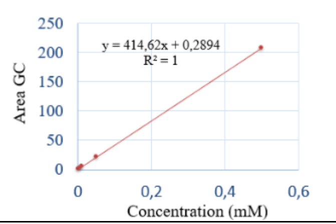	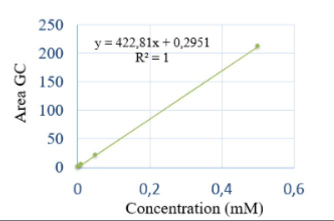
	**11c,C18:1**	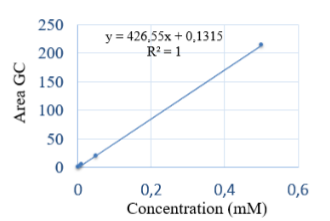	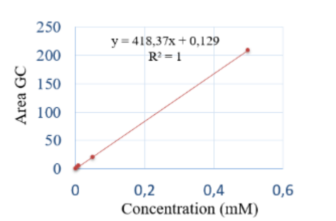	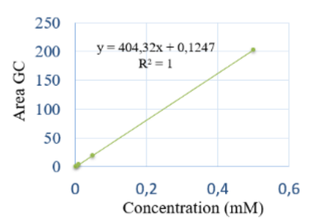
**PUFA omega -3**	**EPA**	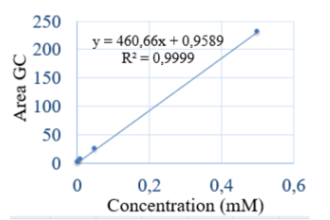	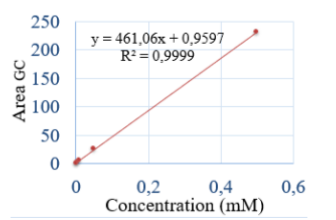	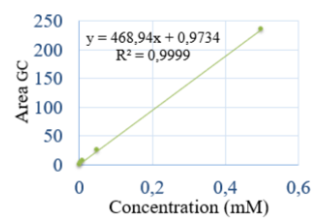
	**DHA**	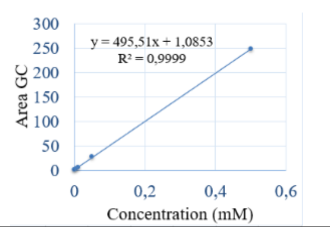	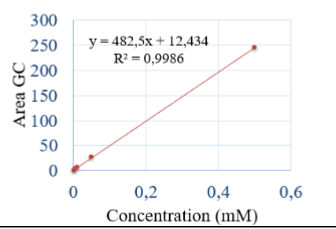	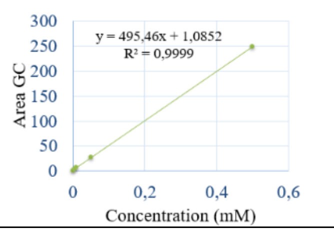
**PUFA omega -6**	**LA**	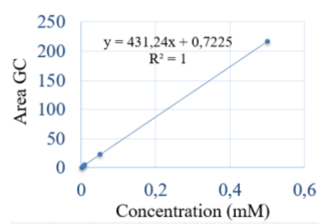	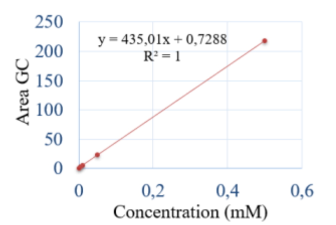	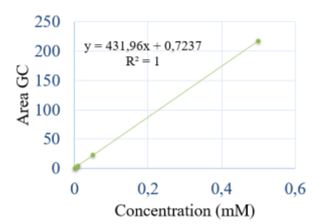
	**DGLA**	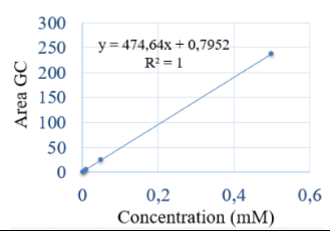	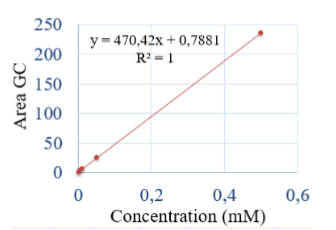	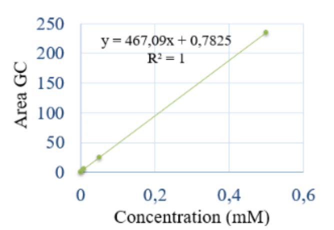
	**ARA**	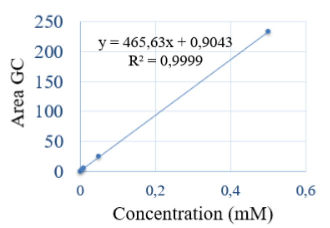	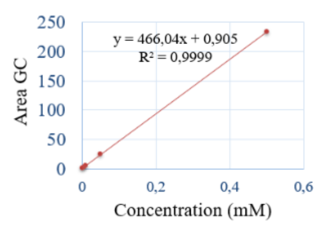	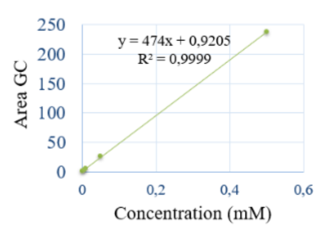

Supplementary Figure 1. Calibration curves of the 10 fatty acids at high (0.5-5 mM) and low (from 0.001 mM to 0.5 mM) concentration ranges, chosen as representatives of the SFA, MUFA and PUFA families present in the erythrocyte membrane phospholipids.

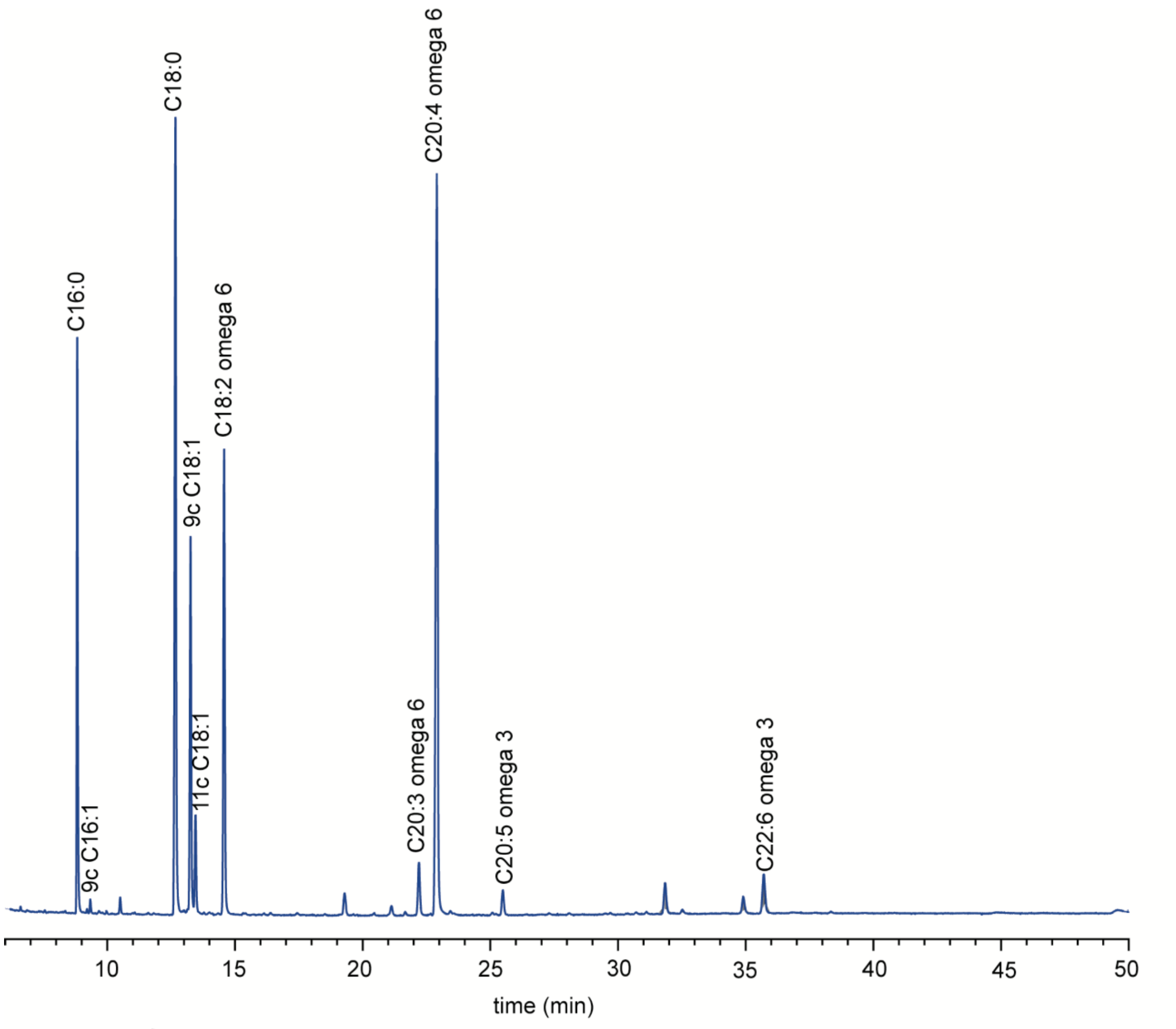

Supplementary Figure 2. Representative GC chromatogram of the FAME obtained from dog erythrocyte membrane phospholipids after work-up, as described in the main text. The 10 fatty acids chosen for the cluster are satisfactorily separated and recognized by appropriate standard references. The sum of their areas corresponds to >97% of the total peak areas.

Fatty acids	Interval values2 (min-max)	Ref. 1	Ref. 2	Ref. 3
Number of samples	68	5	8	30
LA omega-6 – C18:2 – linoleic acid	9.2 - 21	7.80	10.13	-
DGLA omega-6 – C20:3 dihomogammalinolenic acid	0.4 - 2.3	-	1.55	-
ARA omega-6 C20:4 – arachidonic acid	17.5 - 43.7	28.29	28.05	28.6 - 30.8
EPA omega-3 – C20:5 – eicosapentaenoic acid	0.2 - 1.5	-	-	0.364 - 0.42
DHA omega-3 – C22:6 – docosahexaenoic acid	0.2 - 2.5	-	-	0.22 - 0.28
Total SFA	27.8 - 43	-	41.14	-
MUFA	8.7 - 16.7	-	-	-
Total PUFA omega-3	0.5 - 4	-	1.87	1.35 - 1.65
PUFA omega-6	34.9 - 60.8	-	42.31	44.4 - 44.8

Supplementary Table 3. Data of the fatty acids of erythrocyte membranes of our healthy dog cohort (see Table 1 in the main text) together with the data reported in previous literature.

1. Mehta JR, Braund KG, Hegreberg GA, Thukral V. Lipid Fluidity and Composition of the Erythrocyte Membrane for Healthy Dogs and Labrador Retrievers with Hereditary Muscular Dystrophy. *Neurochem Res* (1991) 16:129-35.
2. Fuhrmann H, Zimmermann A, Gück T, Oechtering G. Erythrocyte and plasma fatty acid patterns in dogs with atopic dermatitis and healthy dogs in the same household. *Can J Vet Res* (2006) 70: 191–196.
3. Stoekel K, Nielsen LH, Fuhrmann H, Backmann L. Fatty acid patterns of dog erythrocyte membranes after feeding of a fish-oil based DHA-rich supplement with a base diet low in n-3 fatty acids versus a diet containing added n-3 fatty acids. *Acta Vet Scand* (2011) 53:57. doi: 10.1186/1751-

0147-53-57.

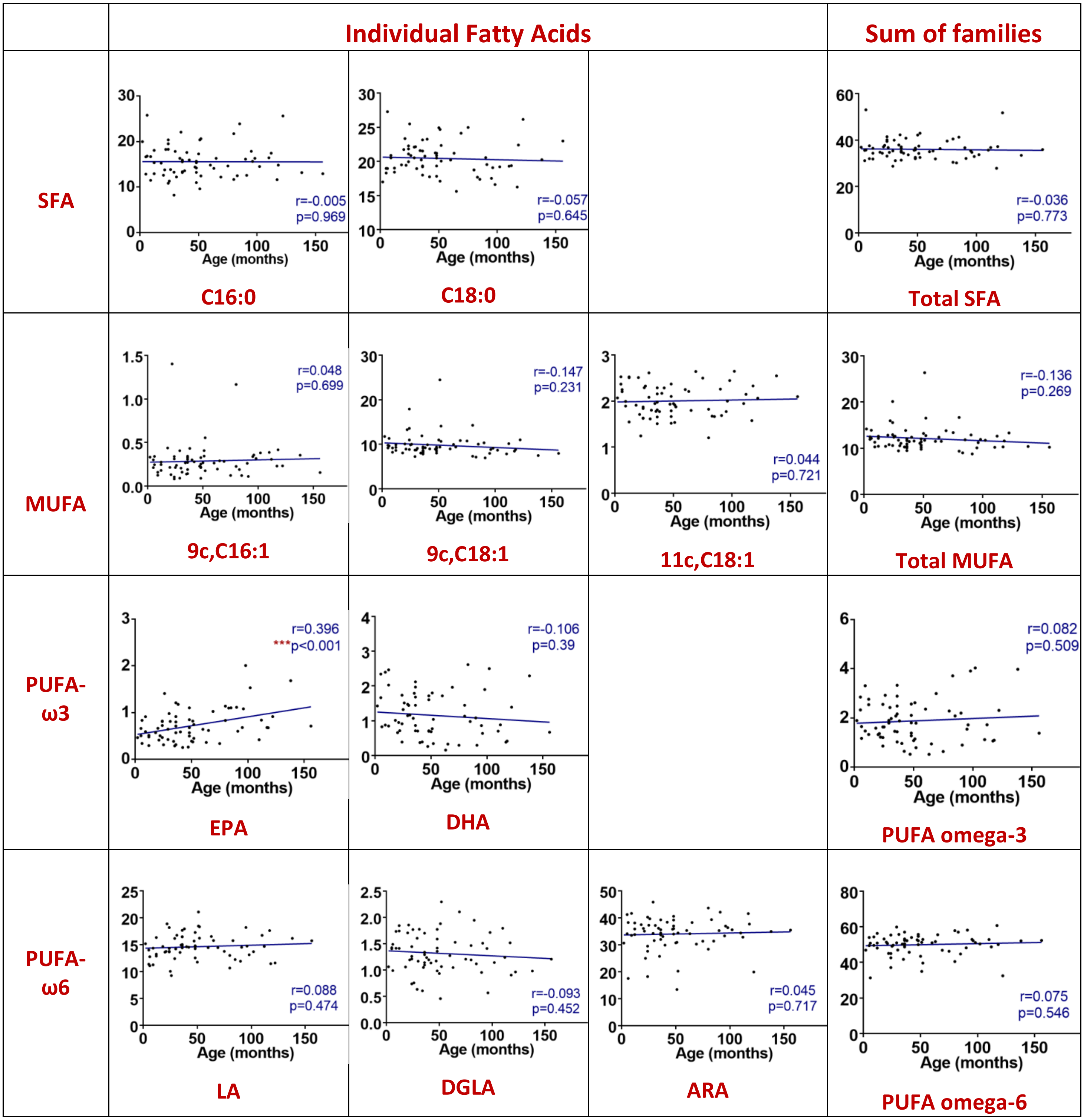

Supplementary Figure 3. Pearson correlation with linear regression and parameters for healthy dogs (n=68) using age and each fatty acid type and family obtained from erythrocyte membranes (data are reported in Tables 1 and 2 in the main text). Each member of the fatty acid family is given in a row, the last column being the sum of the corresponding fatty acid family.

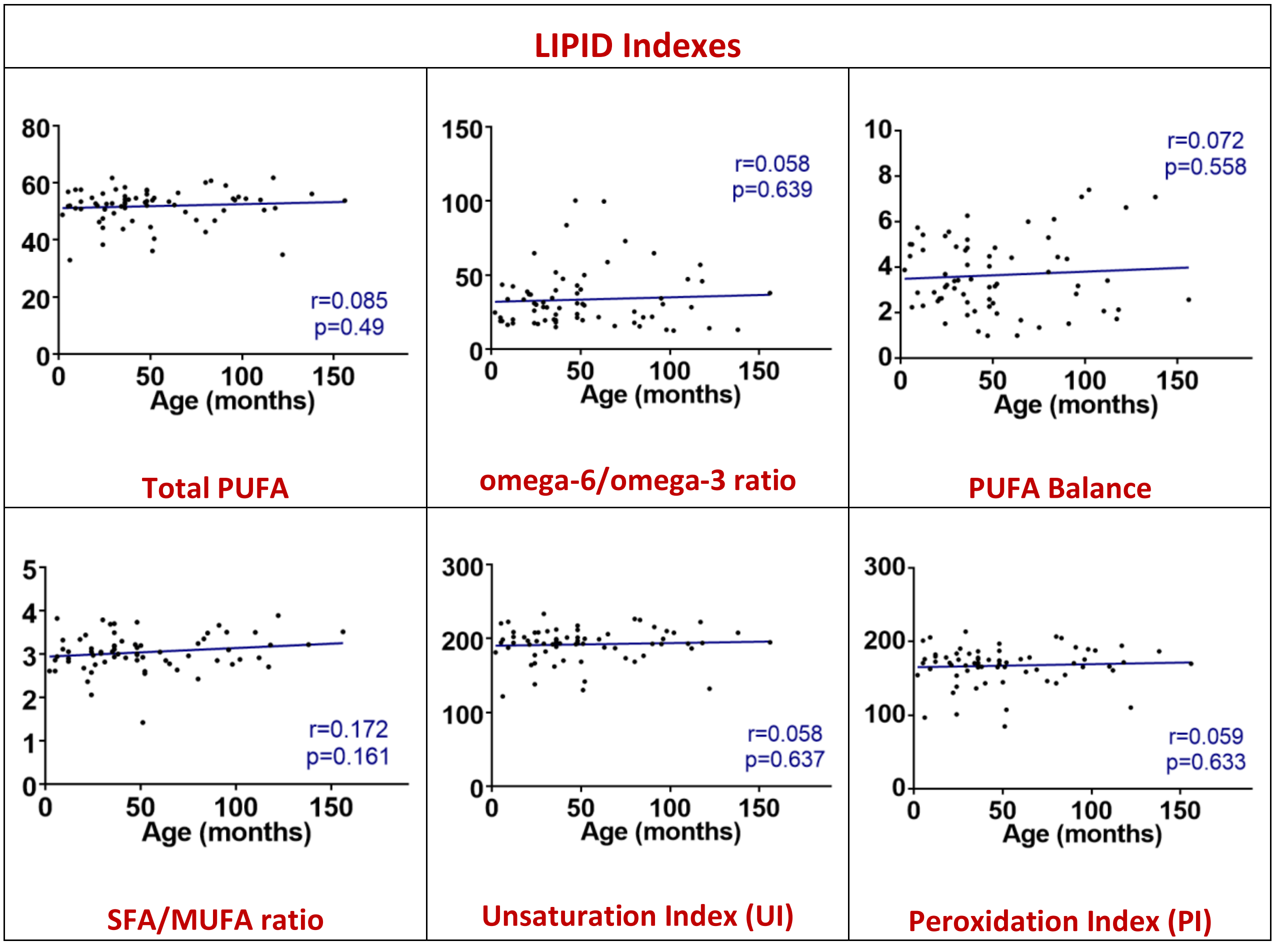

Supplementary Figure 4. Pearson correlation with linear regression and parameters for healthy dogs (n=68) using age and lipid indexes obtained from erythrocyte membranes (data are reported in Tables 1 and 2 in the main text). 1st row: total PUFA, omega-6/omega-3 and PUFA balance ratios; 2nd row: SFA/MUFA ratio, unsaturation and peroxidation indexes.

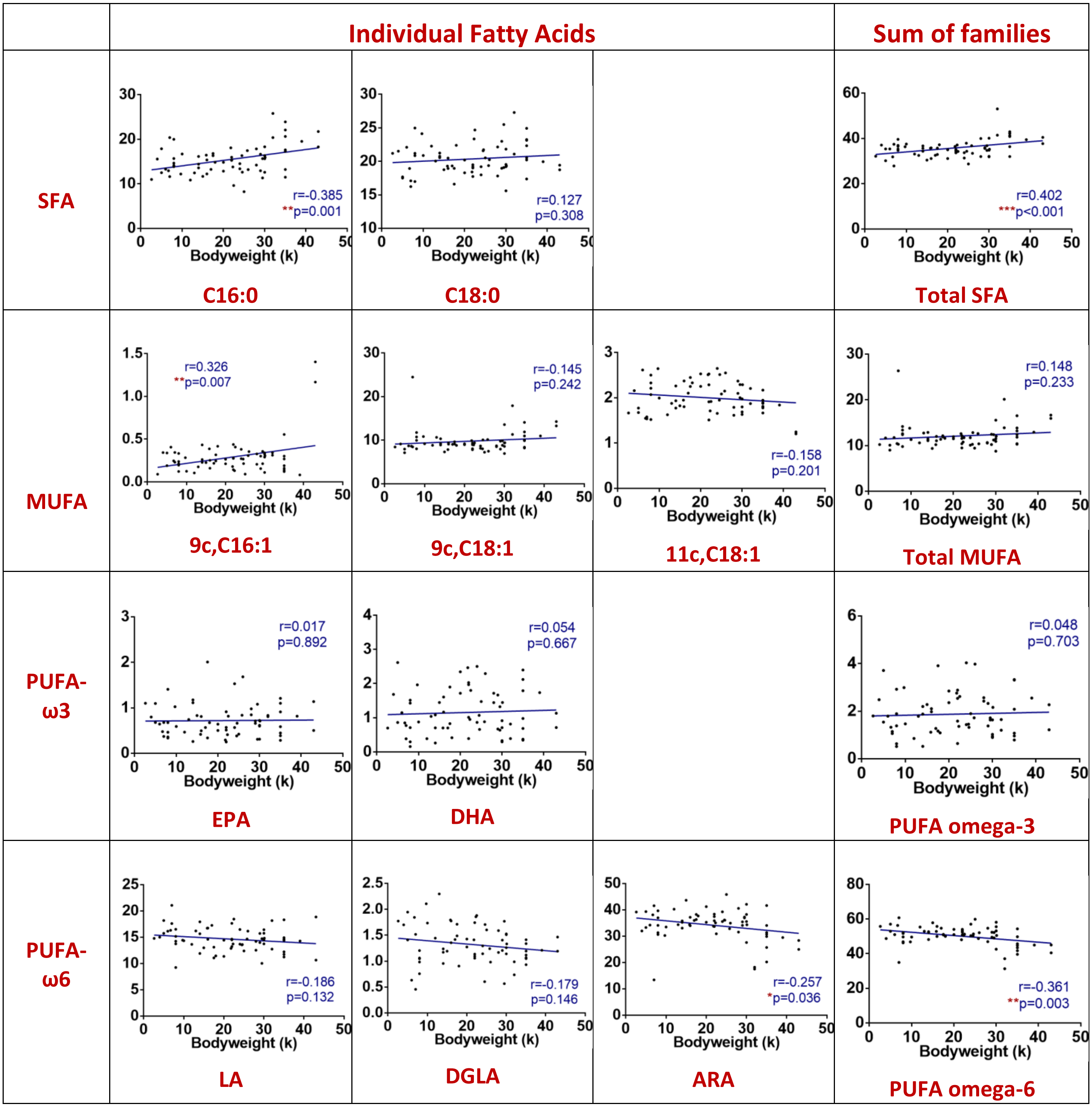

Supplementary Figure 5. Pearson correlation with linear regression and parameters for healthy dogs (n=68) using bodyweight and each fatty acid type and family obtained from erythrocyte membranes (data are reported in Tables 1 and 2 in the main text). Each member of the fatty acid family is given in a row, the last column being the sum of the corresponding fatty acid family.

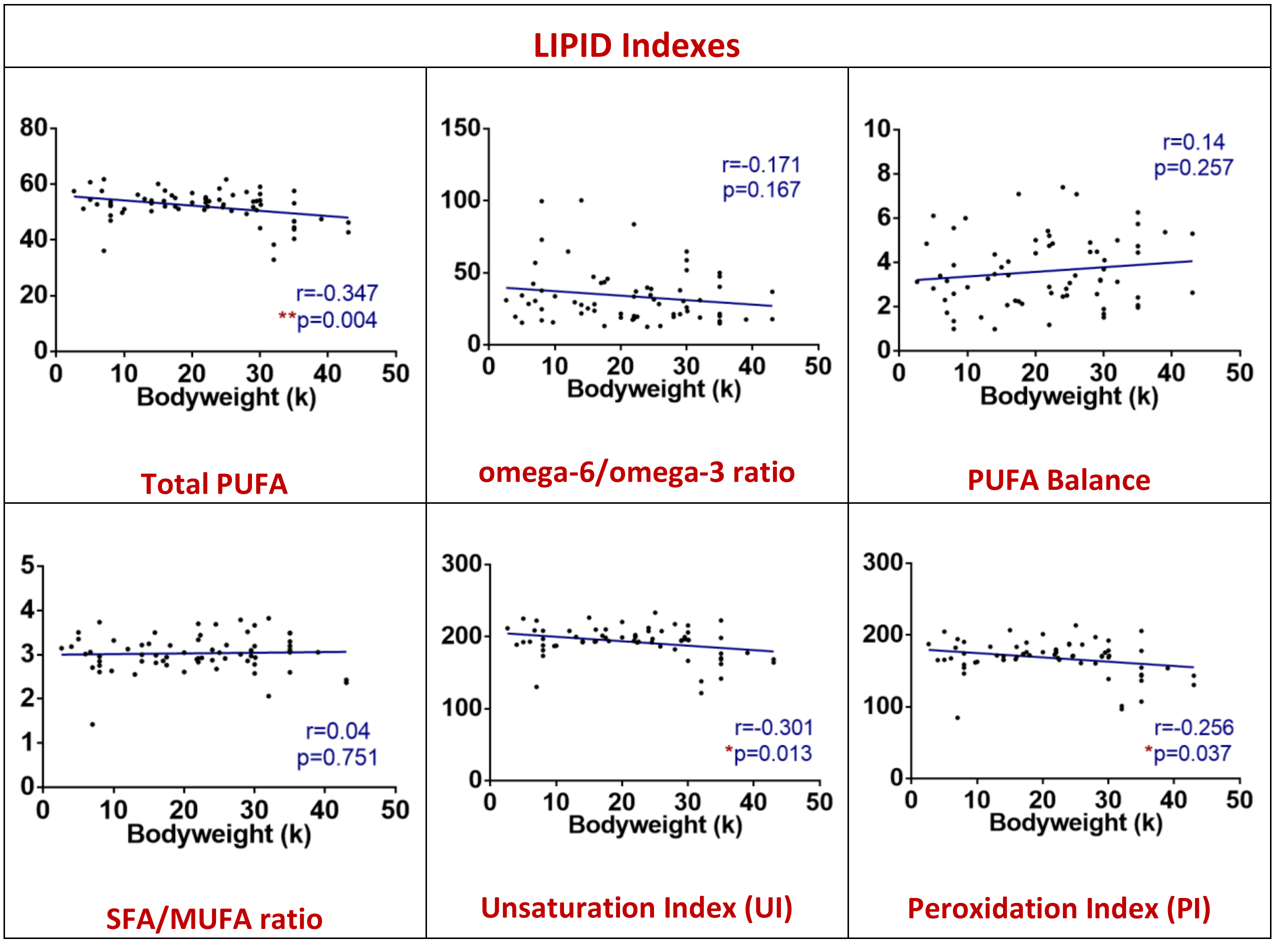

Supplementary Figure 6. Pearson correlation with linear regression and parameters for healthy dogs (n=68) using bodyweight and lipid indexes obtained from erythrocyte membranes (data are reported in Tables 1 and 2 in the main text). 1st row: total PUFA, omega-6/omega-3 and PUFA balance ratios; 2nd row: SFA/MUFA ratio, unsaturation and peroxidation indexes.

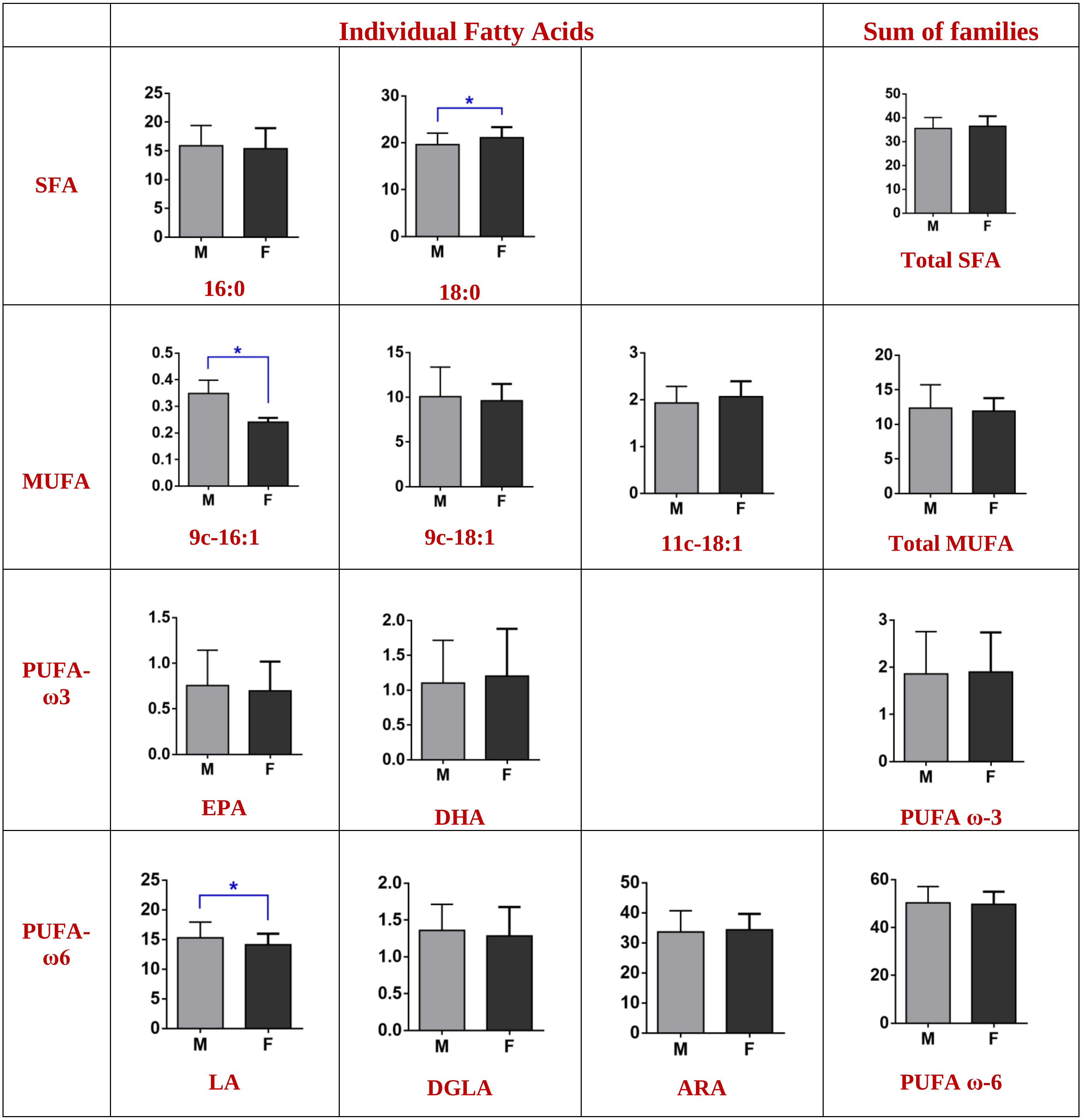

Supplementary Figure 7. Relative quantitative percentage differences between Male (M, grey, n=30) and Female (F, black, n=38) healthy dogs for each type of fatty acid in the erythrocyte membranes. The values are given as mean ± SD. Each member of the fatty acid family is given in a row, the last column being the sum of the corresponding fatty acid family. Values significantly

different when compared to with each other: (*) $p < 0.05$. For healthy dogs' characteristics see Table 1. For specific values see Table 2 in the main text.

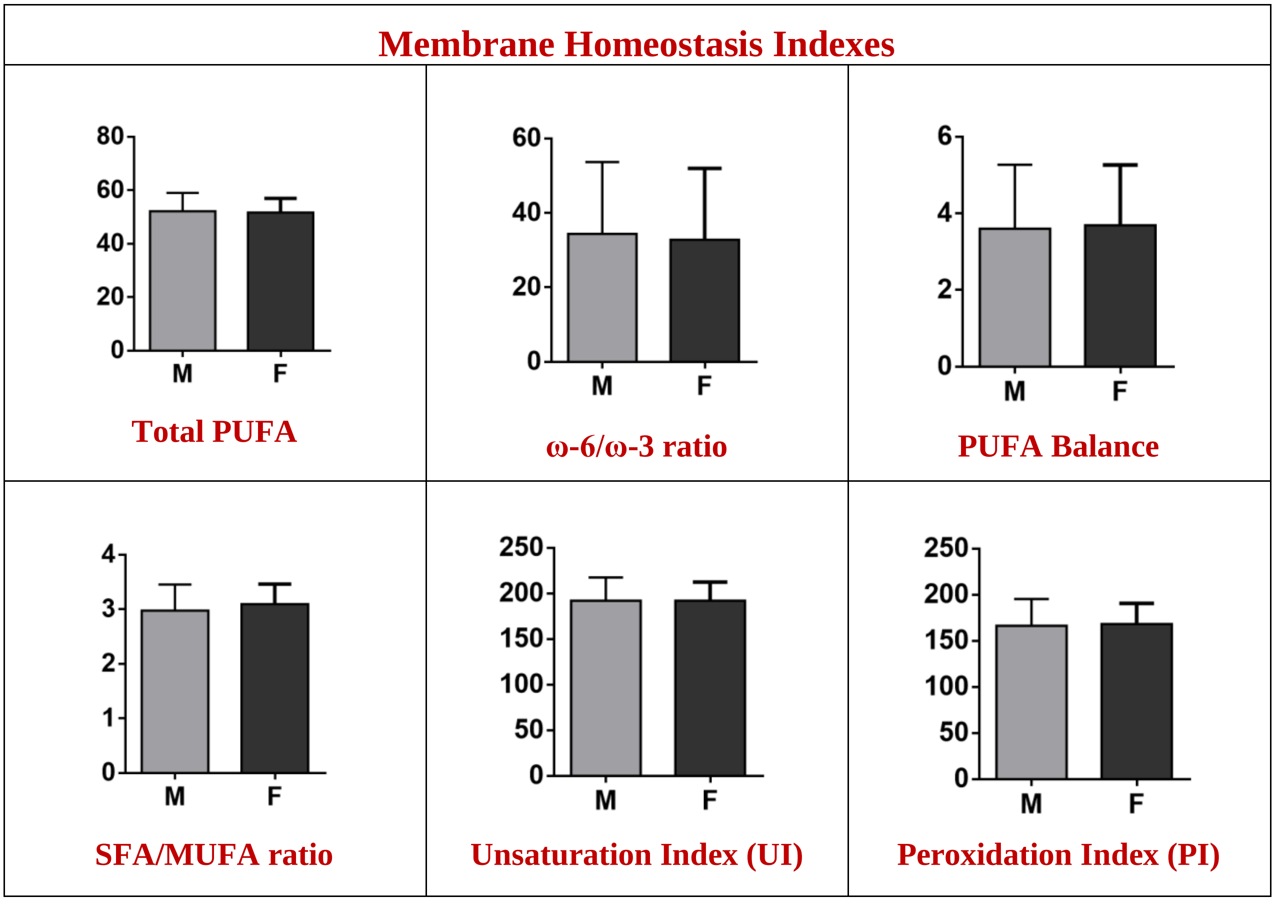

Supplementary Figure 8. Relative quantitative percentage differences between Male (M, grey, n=30) and Female (F, black, n=38) healthy dogs for the membrane homeostasis indexes. 1st row: total PUFA, omega-6/omega-3 and SFA/PUFA ratios; 2nd row: SFA/MUFA ratio, unsaturation and peroxidation indexes. The values are given as mean ± SD. For healthy dog characteristics see Table 1. For specific values see Table 2.
